# Cholecystectomy and subsequent risk of Parkinson’s disease: a nationwide retrospective cohort study

**DOI:** 10.1038/s41531-021-00245-z

**Published:** 2021-11-16

**Authors:** Ryul Kim, Jee-Young Lee, Sanghyun Park, Kyungdo Han, Cheol Min Shin

**Affiliations:** 1 Department of Neurology, Inha University Hospital, Incheon, Korea; 2 Department of Neurology, Seoul National University-Seoul Metropolitan Government Boramae Medical Center, Seoul National University College of Medicine, Seoul, Korea; 3 Department of Biostatistics, College of Medicine, The Catholic University of Korea, Seoul, Korea; 4 Department of Statistics and Actuarial Science, Soongsil University, Seoul, Korea; 5 Department of Internal Medicine, Seoul National University Bundang Hospital, Seongnam, Korea

**Keywords:** Risk factors, Parkinson's disease

## Abstract

Growing evidence has suggested that the gut-brain axis plays an important role in the pathogenesis of Parkinson’s disease (PD), and that this role is mediated by the interactions between bile acids (BAs) and intestinal microbiota. Given that cholecystectomy can lead to alterations in BAs and gut microbiota, we investigated whether cholecystectomy is linked to a higher risk of PD. We constructed a cohort of patients with an operation code of cholecystectomy from 2010 to 2015 (*n* = 161,838) and age- and sex-matched control subjects without cholecystectomy (*n* = 286,135) using the National Health Insurance Service database. Incident PD was traced over a maximum observation period of 7 years. We identified 1404 incident PD cases during 1,631,265 person-years of follow-up. The cholecystectomy group showed an elevated risk of PD compared to the control group, even after adjusting for potential confounding factors (adjusted hazard ratio [HR] 1.14, 95% confidence interval [CI] 1.02–1.27). When the data were split by sex, the risk elevation was significant in men (adjusted HR 1.22, 95% CI 1.06–1.41), but not in women (adjusted HR 1.03, 95% CI 0.88–1.22). Our results provide evidence that cholecystectomy is associated with an increased risk of developing PD. This association differed between men and women, suggesting sex-specific effects of cholecystectomy on the risk of PD.

## Introduction

It has been hypothesized that the gut–brain axis plays an important role in the development of Parkinson’s disease (PD)^1^. Evidence has shown that constipation can precede the onset of motor symptoms of PD by many years^2^. Aggregated α-synuclein, which is a pathologic hallmark of PD, has been observed in the enteric nervous system during the prodromal period^3^. This hypothesis is also supported by epidemiological data showing that truncal vagotomy, which interrupts the gut–brain connection through the vagus nerve, is related to a decreased risk of PD^4,5^. The suggested mechanisms underlying the gut–brain interaction in PD development include an altered gut microbiota, increased intestinal permeability, and intestinal and systemic inflammation^6^. These changes promote α-synuclein aggregation and microglial activation in the brain, resulting in nigrostriatal dopaminergic neurodegeneration^7^.

Cholecystectomy for various gallbladder disorders, including symptomatic gallstones, cholecystitis, and gallbladder tumors, is one of the most commonly performed surgical procedures worldwide^8^. The gallbladder acts as a reservoir and contractile pump of bile acids (BAs), and these compounds are known to interact bidirectionally with the intestinal microbiota^9,10^. Therefore, removal of the gallbladder modifies the enterohepatic circulation of BAs, and subsequently results in changes in microbiota composition and an increase in secondary BAs^8,11^. Recently, the potential role of BAs in various neurodegenerative disorders has been highlighted^12,13^. With respect to PD, previous data have shown that BA profiles are altered in animal models of PD^14,15^, and such changes were associated with gut microbiota dysbiosis in PD patients^16^. These observations are corroborated by genetic association studies, where mutations in the *HSD3B7* gene, which catalyzes the second step of the classical BA synthesis pathway, were linked to PD^17,18^. Interestingly, BAs have shown neuroprotective properties in both animal models and clinical studies of PD^19–21^. In this context, cholecystectomy may contribute to the subsequent risk of PD mainly via changes in BA metabolism; however, the association of cholecystectomy with PD remains unknown.

In this population-based cohort study, we investigated the risk of PD among individuals with and without cholecystectomy. On the basis of previously published data, we hypothesized that cholecystectomy might be associated with a higher incidence of PD.

## Results

### Baseline characteristics

**Fig. 1:**
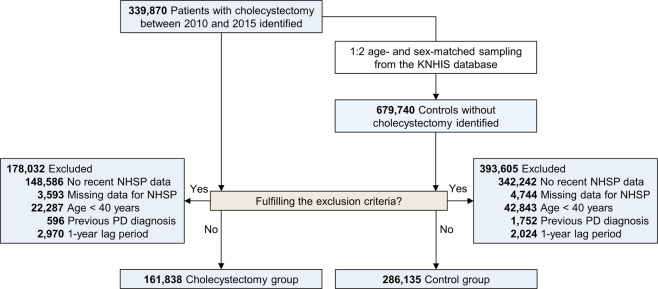
Flow diagram of subject selection. Abbreviations: *KNHIS* Korean National Health Insurance Service, *NHSP* National Health Screening Program, *PD* Parkinson’s disease.

We extracted data on 339,870 patients who underwent cholecystectomy and 679,740 age- and sex-matched control subjects from the Korean National Health Insurance Service (KNHIS) database. After exclusion, a total of 161,838 patients and 286,135 control subjects were included in this study (Fig. 1). Table 1 shows the baseline characteristics of the study population according to the presence of cholecystectomy. The cholecystectomy group had a higher mean body mass index (BMI) and a higher prevalence of diabetes mellitus than the control group (absolute standardized mean difference [ASMD] > 0.1). Subgroups of either sex showed grossly similar baseline characteristics to those of the total cohort of participants (Table 1).

**Table 1:** Baseline characteristics of participants.

	Total			Men			Women		
Variables	Cholecystectomy (*n* = 161,838)	Non-cholecystectomy (*n* = 286,135)	ASMD	Cholecystectomy (*n* = 83,474)	Non-cholecystectomy (*n* = 147,020)	ASMD	Cholecystectomy (*n* = 78,364)	Non-cholecystectomy (*n* = 139,115)	ASMD
Age, years	58.3 (11.2)	58.7 (11.1)	0.044	58.6 (11.1)	59.2 (11.0)	0.046	57.83 (11.2)	58.3 (11.2)	0.043
Male sex	83,474 (52%)	147,020 (51%)	0.004	–	–	–	–	–	–
Smoking			0.029			0.022			0.070
Never smoker	100,998 (62%)	182,567 (64%)		27,046 (32%)	49,180 (33%)		73,952 (94%)	133,387 (96%)	
Ex-smoker	30,085 (19%)	51,426 (18%)		28,728 (34%)	49,622 (34%)		1,357 (2%)	1,804 (1%)	
Current smoker	30,755 (19%)	52,142 (18%)		27,700 (33%)	48,218 (33%)		3,055 (4%)	3,924 (3%)	
Alcohol^a^			0.049			0.080			0.020
Nondrinker	102,732 (63%)	175,047 (61%)		37,443 (45%)	60,107 (41%)		65,289 (83%)	114,940 (83%)	
Moderate drinker	48,583 (30%)	92,335 (32%)		36,092 (43%)	69,118 (47%)		12,491 (16%)	23,217 (17%)	
Heavy drinker	10,523 (7%)	18,753 (7%)		9,939 (12%)	17,795 (12%)		584 (1%)	958 (1%)	
Regular exercise^b^	32,696 (20%)	61,569 (22%)	0.032	19,089 (23%)	36,100 (25%)	0.040	13,607 (17%)	25,469 (18%)	0.025
Low income level^c^	33,281 (21%)	59,898 (21%)	0.009	15,163 (18%)	27,027 (18%)	0.006	18,118 (23%)	32,871 (24%)	0.012
BMI, kg/m^2^	24.6 (3.2)	24.0 (3.1)	0.212	24.7 (3.0)	24.1 (2.9)	0.200	24.6 (3.5)	23.8 (3.2)	0.226
Fasting blood glucose, mg/dl	103.8 (27.2)	101.7 (25.2)	0.081	106.5 (29.2)	104.5 (27.5)	0.072	100.9 (24.7)	98.7 (22.2)	0.092
Serum total cholesterol, mg/dl	195.2 (38.5)	197.1 (37.7)	0.048	190.2 (37.6)	192.9 (37.0)	0.073	200.6 (38.8)	201.4 (37.9)	0.022
History of diabetes mellitus	30,909 (19%)	42,200 (15%)	0.116	18,574 (22%)	25,782 (18%)	0.118	12,335 (16%)	16,418 (12%)	0.115
History of hypertension	70,453 (44%)	113,530 (40%)	0.078	38,812 (47%)	63,018 (43%)	0.073	31,641 (40%)	50,512 (36%)	0.084

Data are *n* (%) and the mean (standard deviation).

^a^Daily amount of alcohol consumption was categorized as follows: heavy drinker, ≥30 g/day; moderate drinker, 1–30 g/day; and nondrinker.

^b^Regular exercise indicates ≥20 min of vigorous-intensity physical activity ≥3 days a week or ≥30 min of moderate-intensity physical activity ≥5 days a week.

^c^Lower income level was defined at the lowest 20%.

Abbreviations: *ASMD* absolute standardized mean difference, *BMI* body mass index.

### Impact of cholecystectomy on PD risk

We identified 1404 cases of incident PD during 1,631,265 person-years of follow-up (Table 2). The PD incidence rate was 0.93 per 1000 person-years among the cholecystectomy group and 0.82 per 1000 person-years in the control group. When the data were divided by sex, men had a higher incidence rate of PD than women. Furthermore, the difference in PD incidence according to cholecystectomy appeared to be greater in men (1.09 per 1000 person-years among the cholecystectomy group vs. 0.90 per 1000 person-years among the control group) than in women (0.77 per 1000 person-years among the cholecystectomy group vs. 0.74 per 1000 person-years among the control group) (Table 2).

**Table 2:** IRs, HRs, and associated 95% CIs for Parkinson’s disease.

	Cholecystectomy	Total no.	Event	Person-years	IR^a^	HR (95% CI)			
						Model 1^b^	Model 2^c^	Model 3^d^	*P*
**Total**									
	Yes	161,838	544	584,566	0.93	1.13 (1.02–1.26)	1.17 (1.05–1.31)	1.14 (1.02–1.27)	0.019
	No	286,135	860	1,046,699	0.82	1	1	1	
**Sex group**									
Men	Yes	83,474	323	297,274	1.09	1.21 (1.05–1.39)	1.26 (1.09–1.45)	1.22 (1.06–1.41)	0.006
	No	147,020	479	532,886	0.90	1	1	1	
Women	Yes	78,364	221	287,292	0.77	1.04 (0.88–1.22)	1.07 (0.91–1.26)	1.03 (0.88–1.22)	0.693
	No	139,115	381	513,813	0.74	1	1	1	
**Age group**									
40–49	Yes	42,148	16	155,297	0.10	1.37 (0.70–2.66)	1.37 (0.70–2.66)	1.39 (0.71–2.73)	0.331
	No	68,302	19	253,016	0.08	1	1	1	
50–59	Yes	49,653	69	182,645	0.38	1.47 (1.07–2.02)	1.47 (1.07–2.02)	1.44 (1.04–1.98)	0.027
	No	86,460	83	322,469	0.26	1	1	1	
60–69	Yes	40,497	193	147,809	1.31	1.29 (1.07–1.54)	1.29 (1.07–1.55)	1.25 (1.04–1.50)	0.017
	No	75,381	284	279,638	1.02	1	1	1	
≥70	Yes	29,540	266	98,815	2.68	1.07 (0.93–1.24)	1.07 (0.93–1.24)	1.04 (0.90–1.20)	0.618
	No	55,992	474	191,576	2.48	1	1	1	

^a^IRs were expressed as per 1000 person-years.

^b^Model 1 was not adjusted (crude).

^c^Model 2 was adjusted for age and sex.

^d^Model 3 was adjusted for age, sex, smoking status, alcohol consumption, regular exercise, income level, body mass index, total serum cholesterol, fasting blood glucose, and the presence of hypertension and diabetes mellitus.

Abbreviations: *CI* confidence interval, *HR* hazard ratio, *IR* incidence rate.

**Fig. 2:**
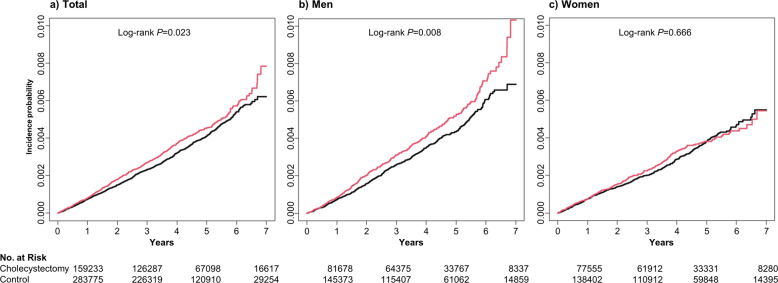
Kaplan–Meier estimates for the probability of incident Parkinson’s disease up to 7 years according to cholecystectomy. **a** Total population; **b** Male population; **c** Female population.

Figure 2 presents the Kaplan–Meier survival curves and log-rank tests for developing PD. In the total population, the cumulative incidence of PD in the cholecystectomy group was significantly higher than that in the control group (*P* = 0.023) (Fig. 2a). This significant difference was prominent in men (*P* = 0.008) (Fig. 2b), but not in women (*P* = 0.666) (Fig. 2c). As shown in Table 2, the crude HR of PD by cholecystectomy in the total population was 1.13 (95% [confidence interval [CI] 1.02–1.26) (Model 1). After adjusting for age, sex, and other covariates (Model 3), the hazard ratio (HR) was 1.14 (95% CI 1.02–1.27; *P* = 0.019) in the cholecystectomy group. Among men, this HR was 1.22 (95% CI 1.06–1.41; *P* = 0.006) and for women it was 1.03 (95% CI 0.88–1.22; *P* = 0.693).

When we divided the participants into four age groups (40–49 years, 50–59 years, 60–69 years, and ≥70 years), a significantly increased risk was observed in the 50–59 years (HR 1.44, 95% CI 1.04–1.98; *P* = 0.027) and 60–69 years group (HR 1.25, 95% CI 1.04–1.50; *P* = 0.017) (Table 2). However, we found no significant risk differences according to cholecystectomy in the 40–49 years and ≥70 years groups.

These results were consistent with those from additional matching (1:1 ratio) data analyses (Supplementary Table 1), as well as with those from analyses with a 2-year lag time (Supplementary Table 2).

### Subgroup analysis

**Fig. 3:**
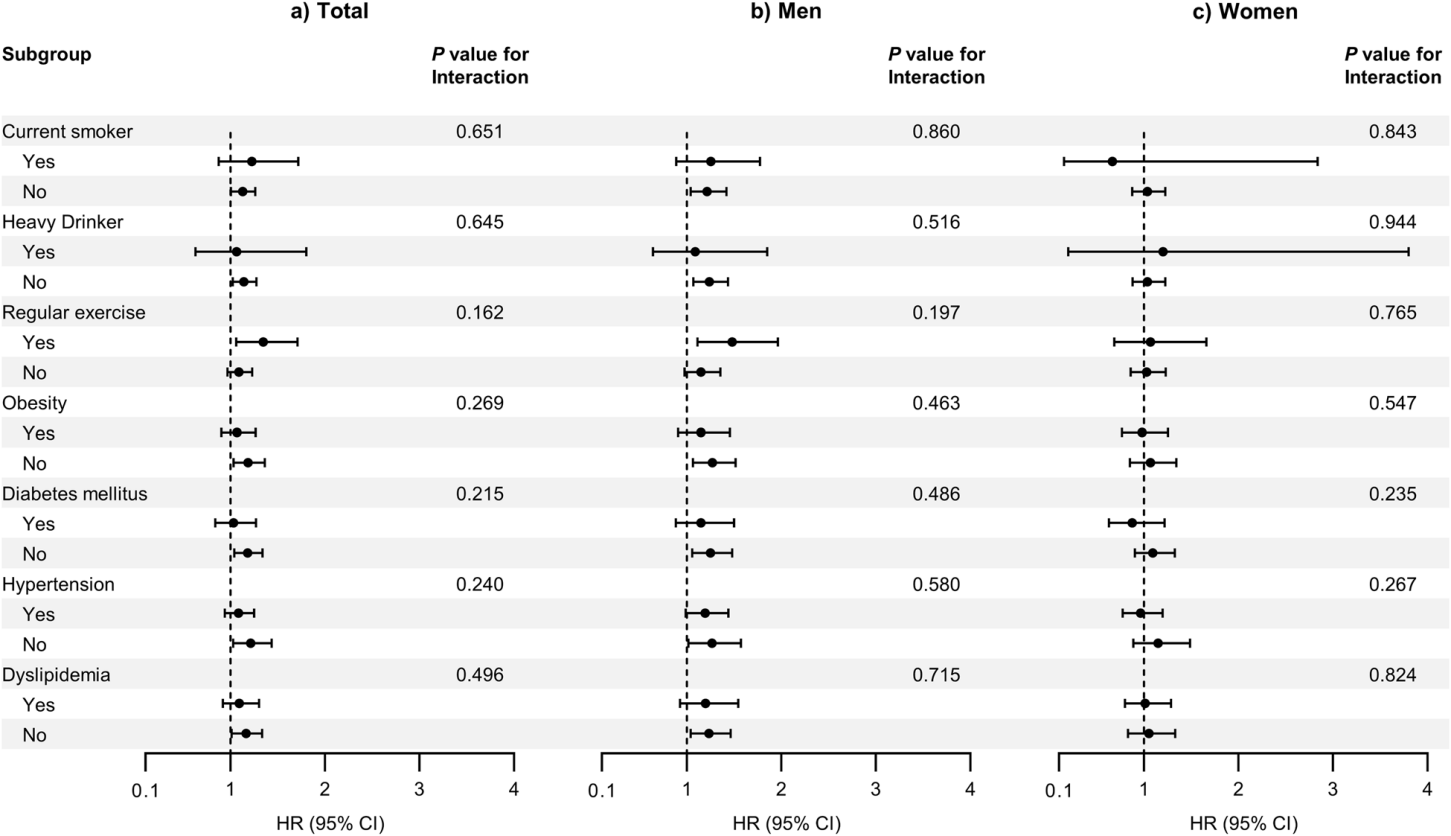
Subgroup analysis of Parkinson’s disease risk in patients with cholecystectomy. **a** Total population; **b** Male population; **c** Female population. Abbreviations: *CI* confidence interval, *HR* hazard ratio.

To assess the effect of modifiers on the association between cholecystectomy and PD risk, we conducted a stratified analysis using several factors including smoking status, alcohol consumption, physical activity, obesity, diabetes mellitus, hypertension, and dyslipidemia (Fig. 3). There was no significant difference in the risk of PD according to the above factors (*P* for interaction >0.05 for all).

## Discussion

Using nationwide cohort data, we evaluated the impact of cholecystectomy on PD development. The present analyses showed that patients who underwent cholecystectomy had a significant elevation in the risk of PD development, and this tendency was prominent in men but not in women. In age-subgroup analyses, an increased risk of PD by cholecystectomy was particularly demonstrated in the 50–59 years and 60–69 years groups. These findings were consistent with the sensitivity analyses.

Previously, Marras et al.^22^ conducted a study in Canada to investigate the association between appendectomy and PD risk, where individuals who underwent cholecystectomy were included as a comparator group. In that study, no significant difference in PD risk was found between individuals with appendectomy and those with cholecystectomy. However, from this result, no conclusion could be drawn concerning the impact of cholecystectomy on PD development because they did not perform a direct comparison between individuals who underwent cholecystectomy and those who did not. In addition, results from observational studies on appendectomy and PD risk are inconsistent^23^. Nevertheless, their data showed that the incidence rate of PD was 40 events per 100,000 person-years in the cholecystectomy group, which was relatively higher than that in the appendectomy (31 events per 100,000 person-years) and control groups (21 events per 100,000 person-years).

While the underlying mechanisms by which cholecystectomy is linked to the risk of PD are not fully understood, an overview of the current knowledge regarding gallbladder and BA physiology may provide valuable insights. The gallbladder is an organ of the digestive system that primarily stores, concentrates, and releases BAs^9^. These actions play an important role in modulating the flow of BAs in enterohepatic circulation and regulating BA composition. A growing body of evidence has suggested a close relationship between BAs and the gut microbiota^24^. BAs can regulate the gut microbiota directly by exerting inhibitory effects or indirectly through the activation of intestinal barrier function, whereas some gut bacteria that produce bile salt hydrolase and 7α-dehydroxylase can modify the BA profile^10^.

Given that gut microbiota dysbiosis is regarded as a contributing element in the pathogenesis of PD^7^, one could speculate that cholecystectomy would increase PD risk by mediating postoperative changes in the intestinal microbiota. Previous reports have shown compositional alterations of gut microbiota following cholecystectomy, although there are some inconsistencies between the studies^25–27^. The cholecystectomy-related changes of gut microbiota shown previously include an increase in the relative abundance of Bifidobacterium and a decrease in Faecalibacterium, Roseburia, and Prevotella^26,27^, which are reported as a gut microbial composition associated with PD^28,29^. In particular, the latter species has also been linked to the progression of motor symptoms in PD^29^ and to isolated rapid eye movement sleep behavior disorder, which is the most potent prodromal marker of PD^30^.

Alternatively, evidence has shown that cholecystectomy leads to an increased proportion of secondary BAs^31,32^. The increase in production of secondary BAs can contribute to impaired metabolic functions mediated by systemic glucose and lipid homeostatic pathways^33^. Supportive studies have revealed a link between cholecystectomy and metabolic syndrome, independent of cholelithiasis^8,11^. Furthermore, patients with Alzheimer’s disease were shown to have significantly lower serum concentrations of a primary BA (cholic acid) and higher concentrations of the bacterially produced, secondary BA (deoxycholic acid), and an increased ratio of deoxycholic/cholic acid was shown to be associated with cognitive decline in these patients^13^. Considering that such metabolic changes could also contribute to the pathomechanisms of PD^34,35^, postoperative changes in secondary BAs may be at least partially responsible for PD development.

One might think that the risk factors for the possible conditions requiring cholecystectomy have altered the true association with the risk of PD. For example, gallstone disease is one of the most common biliary tract diseases, and cholecystectomy is regarded as the primary procedure for the treatment of symptomatic gallstones^36^. Gallstone disease and PD share some important risk factors including age, physical inactivity, obesity, diabetes mellitus, and rapid weight loss^36,37^. To minimize the impact of these confounding factors on our results, we performed further analyses by adding weight change as a covariate in individuals with additional health checkup data within 1 year after the index date. The analyses produced consistent results (Supplementary Table 3), confirming that our observations were not significantly affected by weight change as well as the other factors that were already controlled in the adjusted models. However, we cannot exclude the possibility that unmeasured or unknown confounding factors could have accounted for the relationships observed in this study.

An interesting finding of the present study is that the effect of cholecystectomy on PD risk was significant only in men. Although it could not be completely excluded that the higher incidence of PD in patients with cholecystectomy might be related to an increased contact with the healthcare system around the time of the surgery, this possibility cannot explain such sex-based differences. There are well-known sex differences in PD^38^. Epidemiological data have shown that men are at greater risk of PD than women^39^. The presentation and progression of PD can differ by sex^38^. Similarly, distinct profiles of gut microbiota and BAs have been reported in men and women, and estrogen has been consistently thought to be a biological factor explaining these disparities between the sexes^40,41^. Of note, it has been shown that estrogens directly impact the gut–brain axis^42^. Previous studies on PD have suggested a potential neuroprotective effect of estrogen against nigrostriatal dopaminergic damage via antioxidant, anti‐inflammatory, and anti‐apoptotic pathways^38^. In this framework, estrogen may attenuate the impact of cholecystectomy on PD risk, particularly in premenopausal women. However, this hypothesis is less likely to be significant, considering that the age of the female participants at baseline is mostly beyond the perimenopausal period. There has been no convincing association between other reproductive factors and PD development^37^, and therefore, whether these factors affect our results remains inconclusive. Further studies are necessary to reveal the exact mechanism of sex differences in the impact of cholecystectomy on PD risk.

The current data showed that a significantly elevated risk of PD by cholecystectomy was observed in individuals aged 50–69 years, but not in those aged ≥70 years. However, this observation may not be surprising because aging is the strongest risk factor for developing PD^43^. A nationwide epidemiological study in Korea showed that the incidence of PD increased steeply from age 60–69 years to 70–79 years, and peaked at age 80–89 years^44^. Accordingly, we assume that aging and possibly other environmental exposures for which information was not available in this dataset may contribute to the development of PD more than cholecystectomy in people aged ≥70 years. Our results also showed that there was no significant association between cholecystectomy and PD in individuals aged 40–49 years, which may be explained by the fact that PD rarely occurs before the age of 50 years^44^. In addition, genetic factors are more closely linked to young-onset PD^45^. Further research with a longer follow-up could help to clarify the impact of cholecystectomy on PD risk in this age group.

The major strengths of this study include a large sample size from a population-based cohort, extensive adjustment for potential confounders, and the presence of a matched comparison group. However, there are some limitations in this study. First, the KNHIS database depends on the clinician’s assignment of a diagnostic code for PD. PD diagnosis in clinical practice is not generally confirmed by pathologic confirmation, and thus, there is a possibility of misdiagnosis of PD. For example, atypical parkinsonian disorders such as the parkinsonian subtype of multiple system atrophy or progressive supranuclear palsy with predominant parkinsonism, might be included in a diagnostic code for PD. However, given that these disorders are relatively rare compared to PD, such bias is less likely to be significant in our results. Second, we cannot exclude the possibility that individuals with undiagnosed PD were included at baseline, which might bias the temporal association between cholecystectomy and PD risk. However, we used two different lag periods to reduce this potential bias, and the analyses revealed consistent results. Finally, the reasons for performing cholecystectomy were not available in this dataset, which limits the impact of underlying gallbladder disease on the development of PD.

In conclusion, the current nationwide cohort study revealed that cholecystectomy was associated with an increased risk of PD. This association differed between men and women, suggesting a potential sex difference in the impact of cholecystectomy on PD development. Further research should be performed to validate our findings in other datasets and to investigate the potential role of BAs and their interactions with the gut microbiota in the pathogenesis of PD.

## Methods

### Study design and participants

The KNHIS has launched a compulsory insurance program since 1997 that covers most of the healthcare services implemented in Korea. The KNHIS database stores information on all medical claims, including sociodemographic factors, healthcare utilization, diagnoses, and prescription drugs. Moreover, the KNHIS has been providing a National Health Screening Program (NHSP) at least once every 2 years for the entire population of Korean adults aged 40 years and older. The NHSP includes a self-reported questionnaire on health behaviors, measurements of blood pressure, height, and weight, and blood and urine tests. These data are also stored in the KNHIS database. Details of the standardized method for data collection by the KNHIS have been described elsewhere^46^.

Using the KNHIS database, we created a cohort of patients who underwent cholecystectomy between January 1, 2010 and December 31, 2015. The procedure code for cholecystectomy was gallbladder removal surgery (Q7380). We excluded the following patients: (1) those who had no NHSP data within 2 years before enrollment; (2) those who had missing data for at least one variable of NHSP; (3) those aged under 40 years; (4) those diagnosed with PD before enrollment; and (5) those who developed PD or died during the 1-year lag period. For the control population, age- and sex-matched individuals without cholecystectomy were selected randomly at a 1:2 ratio (two controls per one case) from the KNHIS database. Patients with cholecystectomy and their matched controls were assigned an index date identical to the date of cholecystectomy. The exclusion criteria applied to the control group were the same as those in the cholecystectomy group. All enrolled participants were followed until the date of death or until December 31, 2016.

### Standard protocol approvals, registrations, and patient consents

This study was approved by the Institutional Review Board of Seoul National University Bundang Hospital (IRB number: X-2003/601-904). The requirement for informed consent was waived because the study was based on routinely collected medical claims data.

### Study endpoint

We identified incident PD using the ICD-10 code for PD (G20) in both outpatient visits and hospitalizations. To ensure the validity of PD diagnosis, we confirmed the diagnosis by matching it in the registry system for rare intractable diseases (RIDs) in the KNHIS. The Korean government has implemented a registration program for copayment reduction of up to 90% for this intractable disease, PD. Patients with newly diagnosed PD in Korea are registered to this system, and only neurologists or neurosurgeons can issue the required certification for PD diagnosis. Accordingly, we identified the development of PD using claims data with ICD-10 code G20, confirmed by the RID registration code V124. This method has also been used in many epidemiologic studies of PD in the Korean population^47–49^.

### Covariates

Data regarding age, sex, smoking status, alcohol consumption, physical activity, income level, BMI, fasting blood glucose, total serum cholesterol, and medical history of diabetes mellitus and hypertension were obtained for all participants. All blood tests were performed in a fasting state. Regular exercise was defined as vigorous-intensity physical activity for ≥20 min on ≥3 days per week or moderate-intensity physical activity for ≥30 min on ≥5 days per week. Information on smoking status and alcohol consumption was obtained using a standardized self-administered questionnaire. Participants were classified by smoking status, as nonsmokers, ex-smokers, or current smokers, and by alcohol consumption as nondrinkers, moderate drinkers (less than 30 grams per day), or heavy drinkers (30 or more grams per day)^50^. Low-income level was defined as the lowest 20%.

### Statistical analysis

All statistical analyses were performed using SAS 9.3 (Cary, NC). Baseline characteristics were compared using the ASMD, and a value more than 0.1 indicated a meaningful difference between the study groups^51^. Survival curves were constructed with Kaplan–Meier estimates and compared using the log-rank test. Cox proportional-hazards regression models were used to estimate HRs with 95% CIs for the risk of PD. Model 1 was an unadjusted model (crude); Model 2 was adjusted for age and sex; and Model 3 was further adjusted for smoking status, alcohol consumption, regular exercise, income level, BMI, total serum cholesterol, fasting blood glucose, and the presence of hypertension and diabetes mellitus. To enhance the reliability of the main results, we added two different sensitivity analyses to this study. First, because some participants were unmatched after applying the inclusion/exclusion criteria (Fig. 1), we performed a 1:1 propensity score matching according to age and sex in our dataset. For these matched data, we used stratified Cox proportional-hazards regression models. Second, we performed the same analytic procedure by setting a lag time of 2 years to account for reverse causation. All tests were two-sided and *P* values less than 0.05 were regarded as significant.

### Reporting summary

Further information on research design is available in the Nature Research Reporting Summary linked to this article.

## Supplementary information

Supplementary Information

Reporting Summary

## Data Availability

The datasets for this study are owned by the KNHIS. There are no current sharing agreements, and data are held under a data use contract with the KNHIS.

## Code availability

No previously unreported custom computer code or algorithm was used to generate results in this study.
