# Supplementary material for: Cholecystectomy and subsequent risk of Parkinson’s disease: a nationwide retrospective cohort study
Source: NPJ Parkinsons Dis. 2021 Nov 16;7:100. doi: 10.1038/s41531-021-00245-z (PMC8595409; doi:10.1038/s41531-021-00245-z)
Supplement: Supplementary file 1 — Supplementary Information [file 41531_2021_245_MOESM1_ESM.docx]

**Supplementary Table 1. IRs, HRs, and associated 95% CIs for Parkinson’s disease when using 1:1 propensity score matching**

|  | **Cholecystectomy** | **Total No.** | **Event** | **Person-Years** | **IR^a^** | **HR (95% CI)** | | | | | |
| --- | --- | --- | --- | --- | --- | --- | --- | --- | --- | --- | --- |
|  |  |  |  |  |  | **Model 1^b^** | ***P*** | **Model 2^c^** | ***P*** | **Model 3^d^** | ***P*** |
| **Total** | | | | | | | | | | | |
|  | Yes | 159,679 | 543 | 581,485 | 0.93 | 1.16 (1.02–1.31) | 0.020 | 1.17 (1.04–1.33) | 0.011 | 1.14 (1.01–1.30) | 0.035 |
|  | No | 159,679 | 458 | 569,930 | 0.80 | 1 |  | 1 |  | 1 |  |
| **Sex group** | | | | | | | | | | | |
| Men | Yes | 82,488 | 322 | 295,947 | 1.09 | 1.29 (1.10–1.53) | 0.002 | 1.31 (1.11–1.55) | 0.001 | 1.28 (1.08–1.51) | 0.004 |
|  | No | 82,488 | 245 | 292,042 | 0.84 | 1 |  | 1 |  | 1 |  |
| Women | Yes | 77,191 | 221 | 285,538 | 0.77 | 1.01 (0.84–1.22) | 0.928 | 1.02 (0.84–1.23) | 0.844 | 1.00 (0.82–1.20) | 0.956 |
|  | No | 77,191 | 213 | 277,889 | 0.77 | 1 |  | 1 |  | 1 |  |
| **Age group** | | | | | | | | | | | |
| 40–49 | Yes | 38,280 | 14 | 144,556 | 0.10 | 1.67 (0.70–3.97) | 0.249 | 1.67 (0.70–3.98) | 0.247 | 1.70 (0.71–4.07) | 0.237 |
|  | No | 38,280 | 8 | 139,210 | 0.06 | 1 |  | 1 |  | 1 |  |
| 50–59 | Yes | 48,758 | 64 | 181,107 | 0.35 | 1.52 (1.02–2.24) | 0.038 | 1.52 (1.02–2.24) | 0.037 | 1.47 (0.99–2.19) | 0.055 |
|  | No | 48,758 | 41 | 176,366 | 0.23 | 1 |  | 1 |  | 1 |  |
| 60–69 | Yes | 40,872 | 180 | 148,824 | 1.21 | 1.37 (1.10–1.72) | 0.006 | 1.37 (1.10–1.72) | 0.006 | 1.36 (1.08–1.71) | 0.008 |
|  | No | 40,872 | 129 | 147,040 | 0.88 | 1 |  | 1 |  | 1 |  |
| ≥70 | Yes | 31,769 | 285 | 106,997 | 2.66 | 1.02 (0.87–1.20) | 0.812 | 1.02 (0.87–1.20) | 0.804 | 0.99 (0.84–1.17) | 0.931 |
|  | No | 31,769 | 280 | 107,314 | 2.61 | 1 |  | 1 |  | 1 |  |

^a^IRs were expressed as per 1,000 person-years.

^b^Model 1 was not adjusted (crude).

^c^Model 2 was adjusted for age and sex.

^d^Model 3 was adjusted for age, sex, smoking status, alcohol consumption, regular exercise, income level, body mass index, total serum cholesterol, fasting blood glucose, and the presence of hypertension and diabetes mellitus.

Abbreviations: CI=confidence interval; HR=hazard ratio; IR=incidence rate.

**Supplementary Table 2. IRs, HRs, and associated 95% CIs for Parkinson’s disease when using a 2-year lag period**

|  | **Cholecystectomy** | **Total No.** | **Event** | **Person-Years** | **IR^a^** | **HR (95% CI)** | | | | | |
| --- | --- | --- | --- | --- | --- | --- | --- | --- | --- | --- | --- |
|  |  |  |  |  |  | **Model 1^b^** | ***P*** | **Model 2^c^** | ***P*** | **Model 3^d^** | ***P*** |
| **Total** | | | | | | | | | | | |
|  | Yes | 159,224 | 416 | 424,042 | 0.98 | 1.15 (1.02–1.30) | 0.027 | 1.19 (1.05–1.35) | 0.005 | 1.15 (1.02–1.31) | 0.025 |
|  | No | 283,769 | 650 | 761,715 | 0.85 | 1 |  | 1 |  | 1 |  |
| **Sex group** | | | | | | | | | | | |
| Men | Yes | 81,675 | 254 | 214,699 | 1.18 | 1.23 (1.05–1.44) | 0.012 | 1.28 (1.09–1.50) | 0.003 | 1.24 (1.06–1.46) | 0.009 |
|  | No | 145,370 | 373 | 386,677 | 0.96 | 1 |  | 1 |  | 1 |  |
| Women | Yes | 77,549 | 162 | 209,343 | 0.77 | 1.05 (0.86–1.27) | 0.637 | 1.08 (0.89–1.31) | 0.439 | 1.04 (0.86–1.26) | 0.694 |
|  | No | 138,399 | 277 | 375,038 | 0.74 | 1 |  | 1 |  | 1 |  |
| **Age group** | | | | | | | | | | | |
| 40–49 | Yes | 41,958 | 11 | 113,246 | 0.10 | 1.38 (0.62–3.07) | 0.435 | 1.37 (0.62–3.07) | 0.438 | 1.43 (0.64–3.21) | 0.387 |
|  | No | 68,237 | 13 | 184,746 | 0.07 | 1 |  | 1 |  | 1 |  |
| 50–59 | Yes | 49,154 | 52 | 133,233 | 0..39 | 1.44 (1.00–2.08) | 0.051 | 1.44 (1.00–2.08) | 0.051 | 1.38 (0.95–1.99) | 0.090 |
|  | No | 86,218 | 64 | 236,125 | 0.27 | 1 |  | 1 |  | 1 |  |
| 60–69 | Yes | 39,739 | 152 | 107,695 | 1.41 | 1.33 (1.08–1.64) | 0.007 | 1.33 (1.08–1.64) | 0.007 | 1.29 (1.05–1.59) | 0.016 |
|  | No | 74,782 | 217 | 204,563 | 1.06 | 1 |  | 1 |  | 1 |  |
| ≥70 | Yes | 28,373 | 201 | 69,868 | 2.88 | 1.09 (0.92–1.29) | 0.305 | 1.09 (0.92–1.29) | 0.304 | 1.05 (0.89–1.24) | 0.603 |
|  | No | 54,532 | 356 | 136,281 | 2.61 | 1 |  | 1 |  | 1 |  |

^a^IRs were expressed as per 1,000 person-years.

^b^Model 1 was not adjusted (crude).

^c^Model 2 was adjusted for age and sex.

^d^Model 3 was adjusted for age, sex, smoking status, alcohol consumption, regular exercise, income level, body mass index, total serum cholesterol, fasting blood glucose, and the presence of hypertension and diabetes mellitus.

Abbreviations: CI=confidence interval; HR=hazard ratio; IR=incidence rate.

**Supplementary Table 3. IRs, HRs, and associated 95% CIs for Parkinson’s disease when adding weight change as a covariate**

|  | **Cholecystectomy** | **Total No.** | **Event** | **Person-Years** | **IR^a^** | **HR (95% CI)** | | | | | |
| --- | --- | --- | --- | --- | --- | --- | --- | --- | --- | --- | --- |
|  |  |  |  |  |  | **Model 1^b^** | ***P*** | **Model 2^c^** | ***P*** | **Model 3^d^** | ***P*** |
| **Total** | | | | | | | | | | | |
|  | Yes | 102,528 | 344 | 383,523 | 0.90 | 1.20 (1.05–1.37) | 0.007 | 1.23 (1.07–1.40) | 0.003 | 1.21 (1.06–1.39) | 0.005 |
|  | No | 207,202 | 577 | 773,597 | 0.75 | 1 |  | 1 |  | 1 |  |
| **Sex group** | | | | | | | | | | | |
| Men | Yes | 54,581 | 215 | 201,846 | 1.07 | 1.32 (1.11–1.56) | 0.002 | 1.36 (1.14–1.61) | <0.001 | 1.34 (1.13–1.60) | <0.001 |
|  | No | 108,816 | 326 | 402,685 | 0.81 | 1 |  | 1 |  | 1 |  |
| Women | Yes | 47,947 | 129 | 181,677 | 0.71 | 1.05 (0.85–1.30) | 0.656 | 1.06 (0.86–1.31) | 0.597 | 1.04 (0.84–1.29) | 0.707 |
|  | No | 98,386 | 251 | 370,912 | 0.68 | 1 |  | 1 |  | 1 |  |

^a^IRs were expressed as per 1,000 person-years.

^b^Model 1 was not adjusted (crude).

^c^Model 2 was adjusted for age, sex, smoking status, alcohol consumption, regular exercise, income level, body mass index, total serum cholesterol, fasting blood glucose, and the presence of hypertension and diabetes mellitus.

^d^Model 3 was adjusted for age, sex, smoking status, alcohol consumption, regular exercise, income level, body mass index, total serum cholesterol, fasting blood glucose, the presence of hypertension and diabetes mellitus, and weight change (weight increase [ ≥5%], maintenance [-5% to 5%], and decrease [<-5%]).

Abbreviations: CI=confidence interval; HR=hazard ratio; IR=incidence rate.
