# Supplementary material for: Cholecystectomy and subsequent risk of Parkinson’s disease: a nationwide retrospective cohort study
Source: NPJ Parkinsons Dis. 2021 Nov 16;7:100. doi: 10.1038/s41531-021-00245-z (PMC8595409; doi:10.1038/s41531-021-00245-z)
Supplement: Supplementary file 2 — Reporting Summary [file 41531_2021_245_MOESM2_ESM.pdf]

nature portfolio

Corresponding author(s): J.Y. Lee and C.M. Shin

Last updated by author(s): Jun 28, 2021

Reporting Summary

Nature Portfolio wishes to improve the reproducibility of the work that we publish. This form provides structure for consistency and transparency in reporting. For further information on Nature Portfolio policies, see our Editorial Policies and the Editorial Policy Checklist.

Statistics

For all statistical analyses, confirm that the following items are present in the figure legend, table legend, main text, or Methods section.

n/a	Confirmed	
☐	☒	The exact sample size (n) for each experimental group/condition, given as a discrete number and unit of measurement
☐	☒	A statement on whether measurements were taken from distinct samples or whether the same sample was measured repeatedly
☐	☒	The statistical test(s) used AND whether they are one- or two-sided<br>*Only common tests should be described solely by name; describe more complex techniques in the Methods section.*
☐	☒	A description of all covariates tested
☐	☒	A description of any assumptions or corrections, such as tests of normality and adjustment for multiple comparisons
☐	☒	A full description of the statistical parameters including central tendency (e.g. means) or other basic estimates (e.g. regression coefficient) AND variation (e.g. standard deviation) or associated estimates of uncertainty (e.g. confidence intervals)
☐	☒	For null hypothesis testing, the test statistic (e.g. F, t, r) with confidence intervals, effect sizes, degrees of freedom and P value noted<br>*Give P values as exact values whenever suitable.*
☒	☐	For Bayesian analysis, information on the choice of priors and Markov chain Monte Carlo settings
☒	☐	For hierarchical and complex designs, identification of the appropriate level for tests and full reporting of outcomes
☒	☐	Estimates of effect sizes (e.g. Cohen's d, Pearson's r), indicating how they were calculated

Our web collection on statistics for biologists contains articles on many of the points above.

Software and code

Policy information about availability of computer code

Data collection	No software was used.
Data analysis	SAS 9.3 (Cary, NC) was used.

For manuscripts utilizing custom algorithms or software that are central to the research but not yet described in published literature, software must be made available to editors and reviewers. We strongly encourage code deposition in a community repository (e.g. GitHub). See the Nature Portfolio guidelines for submitting code & software for further information.

Data

Policy information about availability of data

All manuscripts must include a data availability statement. This statement should provide the following information, where applicable:

- Accession codes, unique identifiers, or web links for publicly available datasets
- A description of any restrictions on data availability
- For clinical datasets or third party data, please ensure that the statement adheres to our policy

The datasets for this study are owned by the Korean National Health Insurance Service (KNHIS). There are no current sharing agreements, and data are held under a data use contract with the KNHIS.

March 2021

Field-specific reporting

Please select the one below that is the best fit for your research. If you are not sure, read the appropriate sections before making your selection.

☒ Life sciences ☐ Behavioural & social sciences ☐ Ecological, evolutionary & environmental sciences

For a reference copy of the document with all sections, see nature.com/documents/nr-reporting-summary-flat.pdf

Life sciences study design

All studies must disclose on these points even when the disclosure is negative.

Sample size	We extracted data on 339,870 patients with cholecystectomy and 679,740 age- and sex-matched comparison subjects from the Korean National Health Insurance Service (KNHIS) database. After exclusion, a total of 161,838 patients and 286,135 comparison subjects were finally included in this study.
Data exclusions	We excluded the following patients: (1) those who had no National Health Screening Program (NHSP) data within 2 years before the enrollment; (2) those who had missing data for at least one variable of NHSP; (3) those aged under 40 years (4) those diagnosed with PD before the enrollment; and (5) those who developed PD or died during 1-year lag period. The exclusion criteria applied to the control group were identical to those of the cholecystectomy group.
Replication	We did not perform a replication study.
Randomization	Not applicable, this is an observational study.
Blinding	Not applicable, this is an observational study.

Reporting for specific materials, systems and methods

We require information from authors about some types of materials, experimental systems and methods used in many studies. Here, indicate whether each material, system or method listed is relevant to your study. If you are not sure if a list item applies to your research, read the appropriate section before selecting a response.

Materials & experimental systems

n/a	Involved in the study	
☒	☐	Antibodies
☒	☐	Eukaryotic cell lines
☒	☐	Palaeontology and archaeology
☒	☐	Animals and other organisms
☐	☒	Human research participants
☒	☐	Clinical data
☒	☐	Dual use research of concern

Methods

n/a	Involved in the study	
☒	☐	ChIP-seq
☒	☐	Flow cytometry
☒	☐	MRI-based neuroimaging

Human research participants

Policy information about studies involving human research participants

Population characteristics	In this study, we reported the following population characteristics: age, sex, smoking status, alcohol consumption, physical activity, income level, body mass index (BMI), fasting blood glucose, total serum cholesterol, and medical history of diabetes mellitus and hypertension. Compared with the control group, the cholecystectomy group had a higher mean BMI and a higher prevalence of diabetes mellitus. Subgroups with either men or women showed baseline characteristics grossly similar to those of the total cohort of participants.
Recruitment	The Korean National Health Insurance Service (KNHIS) has launched compulsory insurance program since 1997 that covers most of the healthcare services implemented in Korea. The KNHIS database stores information on all medical claims, including sociodemographic factors, healthcare utilization, diagnoses, and prescription drugs. Moreover, the KNHIS has been providing a National Health Screening Program (NHSP) at least once every 2 years for the entire population of Korean adults aged 40 years and older. The NHSP includes a self-reported questionnaire on health behaviors, measurements of blood pressure, height, and weight, and blood and urine tests. These data are also stored in the KNHIS database. Using the KNHIS database, we created a cohort of patients who underwent cholecystectomy between January 1, 2010 and December 31, 2015. The procedure code for cholecystectomy was gallbladder removal surgery (Q7380). For the control population, we randomly selected age- and sex-matched individuals without cholecystectomy as controls at a 1:2 ratio (one case for two controls).
Ethics oversight	This study was approved by the Institutional Review Board of the Seoul National University Bundang Hospital (IRB number:

Ethics oversight	X-2003/601-904). The requirement for informed consent was waived because the study was based on routinely collected medical claim data.

Note that full information on the approval of the study protocol must also be provided in the manuscript.
